# Innovations in structure-based antigen design and immune monitoring for next generation vaccines

**DOI:** 10.1016/j.coi.2020.03.013

**Published:** 2020-08

**Authors:** Andrew B Ward, Ian A Wilson

**Affiliations:** 1 Department of Integrative Structural and Computational Biology, The Scripps Research Institute, La Jolla, CA 92037, USA; 2 Scripps Consortium for HIV/AIDS Vaccine Development (CHAVD), The Scripps Research Institute, La Jolla, CA 92037, USA; 3 IAVI Neutralizing Antibody Center and the Collaboration for AIDS Vaccine Discovery (CAVD), The Scripps Research Institute, La Jolla, CA 92037, USA; 4 Skaggs Institute for Chemical Biology, The Scripps Research Institute, La Jolla, CA 92037, USA

## Abstract

The recent explosion of atomic-level structures of glycoproteins that comprise the surface antigens of human enveloped viruses, such as RSV, influenza, and HIV, provide tremendous opportunities for rational, structure-based vaccine design. Several concepts in structure-based vaccine design have been put into practice and are are well along preclinical and clinical implementation. Testing of these designed immunogens will provide key insights into the ability to induce the desired immune responses, namely neutralizing antibodies. Many of these immunogens in human clinical trials represent only the first wave of designs and will likely require continued tweaking and elaboration to achieve the ultimate goal of enhanced breadth and potency. Considerable effort is now being invested in germline targeting, epitope focusing, and improved immune presentation such as multivalent nanoparticle incorporation. This review highlights some of the recent advances in these areas as we prepare for the next generation of immunogens for subsequent rounds of iterative vaccine development.

## Highlights

• Immunofocusing as a strategy for structure-based vaccine design.
• Structure-based vaccine design for RSV F, influenza HA, HIV-1 Env.
• Structure-based vaccine design translated into the clinic.

**Current Opinion in Immunology** 2020, **65**:50–56

This review comes from a themed issue on **Vaccines**

Edited by **Bali Pulendran** and **Rino Rappuoli**

For a complete overview see the Issue and the Editorial

Available online 22nd April 2020

**https://doi.org/10.1016/j.coi.2020.03.013**

0952-7915/© 2020 The Author(s). Published by Elsevier Ltd. This is an open access article under the CC BY license (http://creativecommons.org/licenses/by/4.0/).

## Introduction

The elicitation of neutralizing antibodies is a strong correlate of successful vaccines. These antibodies typically target an essential component of the viral lifecycle, such as the surface glycoproteins of enveloped viruses that enable cell entry through binding to host receptors and facilitating membrane fusion. Such viral antigens include influenza virus hemagglutinin, human immunodeficiency virus (HIV) envelope, and respiratory syncytial virus (RSV) F. Passive administration of antibodies that target these proteins, such as HIV broadly neutralizing antibodies (bnAbs) have proven effective in suppression of viremia in HIV-infected individuals [1,2^•^,^3^] [NCT02825797]. These studies provide proof-of-concept that, if similar antibodies could be induced by a vaccine with sufficient potency, breadth and half-life, individuals could be protected from disease acquisition. A large clinical trial, known as AMP, is currently ongoing to test such a concept in HIV prevention, which will provide important benchmarks in terms of the titers and potency required to block transmission of HIV [NCT02716675 and NCT02568215, AMPstudy.org].

**Figure 1:**
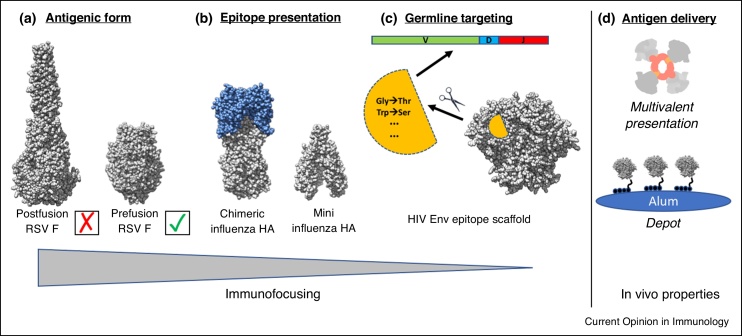
Overview of the different aspects of structure-based antigen design. Because many of the targets being pursued are envelope glycoproteins that mediate viral entry into the cell, they are metastable by nature and adopt different conformations to carry out their function. **(a)** In the case of RSV F, many mutations have now been introduced to stabilize the prefusion conformation, which is the preferred antigenic state that can be targeted by potent neutralizing antibodies. **(b)** For influenza HA, the prefusion conformation is relatively stable, except at the low pH for membrane fusion, but many potential epitopes are less desirable or result in strain-dependent neutralizing antibody responses that are easily escaped. One strategy to focus on the more conserved HA stalk region is to eliminate the more variable HA head altogether, or make chimeric HA with a conserved stalk attached to variable heads for which there is no pre-existing immunity. **(c)** Many HIV Env bnAbs are restricted in their gene usage and place further constraint on vaccine design. One approach to overcome this hurdle is known as germline targeting in which an epitope is presented on a small scaffold and mutations introduced to bind very specifically, and with high affinity, to a particular germline B cell precursor. **(d)** While there has been much success in structure-based antigen design, protein subunit antigens are not necessarily very immunogenic on their own. Thus, many efforts are ongoing to understand how the immune system responds to protein subunit vaccines and development of new modalities for delivery and formulation, such as multivalent nanoparticles and slow release of antigen, as with alum depots.

The remarkable progress in isolating antibodies from natural infection or immunization using functional B-cell screening or antigen specific B-cell isolation technologies [4, 5, 6, 7] has led to accelerated progress and advances in many fields and enabled the structural definition of key epitopes on viral surface antigens [8]. These structures have in turn enabled reverse vaccinology [9]. While many parallel pursuits are ongoing on a wide variety of viral and microbial pathogens, for the purposes of this review, we will focus here on RSV, influenza, and HIV, which have been the flagship pathogens for structure-based vaccine design and nicely highlight the different goals of the rational design process: immunofocusing, cross-reactivity, germline targeting, and somatic hypermutation. Our review highlights how each of these desired outcomes are being incorporated into the design of immunogens for the aforementioned pathogens (Figure 1).

## Immunofocusing

The concept of immunofocusing can be simply described as the maximization of on-target antibody responses to desired epitope(s) and minimization of off-target antibody responses. For many widely employed vaccines, the induction of appropriate antibodies with high potency and durability is readily accomplished when humans are immunized with inactivated viruses or virus like particles (VLPs). However, for RSV F, VLPs do not induce the desired antibody responses, at least not to levels sufficient for protection. The primary hurdle in RSV is the large abundance of RSV F in its post-fusion conformation on the surface of VLPs. Antibodies that recognize this conformation are easily induced but are not protective, thereby acting as a distraction or competitor for efficacious responses to the prefusion conformation of RSV F. A potential solution to this problem came from stabilization of the prefusion conformation of RSV F, a feat that was accomplished by McLellan *et al.* in 2013 [10]. This stabilized form of the antigen preferentially induces neutralizing antibodies to the appropriate prefusion epitopes and provides an important proof-of-concept, which is currently being tested in clinical trials as a subunit vaccine [11^•^], NCT03049488]. Characterization of antibodies induced to prefusion RSV F, along with their corresponding structures, will reveal the diversity in antibody responses and the most critical epitopes for protection, thereby enabling further opportunities for immunogen design aimed at focusing the antibody response even further. Similarly, the HIV envelope glycoprotein (Env) stabilized in its prefusion conformation is being tested in human clinical trials [NCT03699241, NCT04177355, NCT03783130]. This approach successfully induced a protective neutralizing antibody response in macaques to a single strain of HIV-1, demonstrating another important proof-of-concept that vaccine-induced antibodies can be protective against repeated viral challenge [12^•^]. Here, as with the seasonal influenza vaccine, the induced antibodies are quite narrow and strain specific. Thus, the stabilized prefusion conformations of Env and HA alone are not sufficient to induce broad immunity.

## Cross-reactivity (neutralization breadth)

While the key for RSV F is to induce antibodies to conserved and readily accessible epitopes present on the prefusion conformation, further immunofocusing to one or more specific sites on the surface of a prefusion glycoprotein will be required to generate cross-reactive, broad immunity for more variable viruses, such as influenza and HIV. Here, antibodies must hone in on specific conserved, functional epitopes that are dispersed amidst surfaces that are variable and highly glycosylated. The large diversity of circulating HIV strains and the seasonal and zoonotic antigenic drift in influenza present enormous hurdles for achieving such neutralization breadth. The influenza hemagglutinin (HA) stem is a highly conserved region, although less accessible compared to the immunogenic head region on this surface antigen for which broadly neutralizing antibodies (bnAbs) have been discovered [13, 14, 15, 16, 17,8,18,19]. These findings provided some hope that, with appropriately designed immunogens, such bnAbs can be elicited by vaccination. Here, several concepts are being tested to achieve such cross-reactivity including headless or mini-HAs that dispense with the much more immunogenic head domain and present only the stem epitope [18, 19, 20] [NCT03814720], as well as head-swapped chimeric immunogens that vary the HA head (usually zoonotic HAs that have not been seen by humans so as to reduce/eliminate memory recall responses), but keep the stem the same in attempts to boost stalk-specific responses [21^••^] [NCT03300050]. There are also ongoing efforts to broaden immune responses against the conserved receptor binding site within the more variable influenza HA head. A recently described strategy wherein HAs from eight different strains were presented on a single mosaic VLP effectively promoted cross-reactive responses against the receptor binding domain [22^••^].

The fusion peptide (FP) in HIV-1 Env is highly conserved and has recently emerged as a target epitope with bnAbs being identified that are capable of broadly neutralizing diverse viruses (e.g. PGT151, VRC34, ACS202) [23, 24, 25, 26]. In HIV-1 Env, the FP is much more accessible to antibodies compared to other viral glycoproteins, such as the HA, where the FP is deeply buried inside the structure in the prefusion state. One approach to focus the response on the FP is through repeated immunization with 10−100 s of copies of the FP displayed on carrier proteins, such as keyhole limpet hemacyanin (KLH), followed by a booster immunization with the Env trimer spike to present the FP in a more native context. This approach has produced encouraging responses in animals with up to 31% and 59% neutralization breadth in mice and macaques, respectively, against panel of diverse HIV isolates [27,28^••^]. This concept is now being developed for human clinical trials [29].

## Germline targeting

While immunofocusing on highly conserved epitopes may be sufficient in some cases to elicit cross-reactive bnAbs, not all antibodies are necessarily endowed with such an ability to be matured along a pathway to become bnAbs capable of neutralizing diverse viruses. Thus, researchers are testing the concept of activating particular germline B-cell precursors that are known to have such potential. This concept is most advanced for HIV where it has been shown that bnAbs often have very strict requirements, such as particular germline usage (V, D or J), long or short complementarity determining regions (CDRs), particular heavy-light chain pairing, and so on. For the VRC01 class of CD4 receptor binding site (CD4bs) bnAbs, such requirements have been well defined including VH1-2 gene usage and light chains with a short 5aa LCDR3 (compared to the normal length of 8aa). The Schief lab has developed an immunogen, eOD, that presents the CD4 binding site epitope and have evolved it to bind VRC01-class antibody germline precursors [30, 31, 32]. In KI mice, this immunogen has been shown to stimulate such precursors and enrich for antibodies that have the appropriate properties. It was also demonstrated that the B cell precursor frequency and binding affinity to the eOD immunogen were critical determinants of successful expansion in germinal centers [33^•^]. This very well characterized and parameterized eOD concept is now being tested in humans with results expected in mid-to-late 2020 [NCT03547245]. Here, because VRC01-class antibody signatures are well defined, next generation sequencing (NGS) will be a critical tool in determining if the vaccine has induced and expanded the desirable precursors in the healthy human volunteers. Production and characterization of promising antibodies based on NGS data, coupled to three-dimensional structures in complex with the eOD immunogen, will then enable further improvements to the immunogen and inform design of boosting immunogens to further mature toward VRC01 responses.

Such germline targeting strategies are now being extended to additional epitopes on Env including the N332/V3 supersite that is targeted by many antibodies, such as a more recent bnAb BG18 [34^••^]. Here the problem becomes even more difficult due to requirements for long HCDR3 loops with junctional diversity that is not easy to control for eliciting particular HCDR3 sequences that are known to bind this composite glycan/protein epitope. One important requisite for such research is profiling human antibody repertoires to identify and quantitate bona fide human B cell precursors in the human population, which is now an emerging field [35^••^,36^••^]. Deep sequencing of human antibody repertoires therefore serves multiple purposes: validation of the germline targeting approach, identification of appropriate precursors for vaccine design, and benchmarks for the success of an immunogen to select for and enrich appropriate B cells upon immunization. Because animals each have unique B cell repertoires, mice with human B cells expressing bnAb germline heavy and/or light chains have emerged as a critical model for assessing and improving germline-targeting immunogen strategies [37,38]. This model continues to be improved and knock-in mice can now be rapidly generated using CRISPR/Cas9 [39].

## Somatic hypermutation

Even with the ability to immunofocus on conserved epitopes with appropriate germline B-cells, bnAbs usually require a large amount of somatic hypermutation to acquire the range of properties that enable broad cross-reactivity. During active infection, particularly for HIV-1, constant antigen exposure and continuous antigenic drift are natural drivers for high SHM. In the context of a protein subunit vaccine, the ability to drive SHM remains a much larger hurdle. Another issue is that key mutations for development of breadth are not found at traditional AID hotspots and these have been coined improbable mutations that represent hurdles in bnAb development [40^•^,^41^]. Potential prime boost strategies can however ‘shepherd’ SHM toward bnAbs, and proof-of-concept was demonstrated a number of years ago [42,43]. More recent effort is being placed on the delivery to, and persistence of antigen in, germinal centers to further promote somatic hypermutation. Continuous delivery of antigen [44^••^,45^••^], multivalent antigen presentation [46^•^,47^•^], adjuvants, and other technologies, including immune checkpoint modultation to promote germinal center activity, [48] may help overcome the limitations of protein subunits as immunogens. Many of these concepts must now be tested empirically. Finally, nucleic acid delivery of immunogens, particularly by mRNA, is emerging as a platform that has the potential to speed up immunogen testing and potentially reduce manufacturing costs [49].

## New tools in immune monitoring to enable rapid iteration and pre-clinical work

While immunogen design is being conducted with atomic-level precision, experimental vaccines must be tested in animals and eventually humans, whose immune repertoires are inherently different and heterogeneous. Even amongst individuals in a species, there is a large diversity in the B-cell repertoire that is the result of genetics, immune history, and regular B-cell turnover. Campaigns to sequence antibody-omes are beginning to dissect this diversity and may inform on the genetic potential of humans to respond to designer immunogens [35^••^,36^••^]. Understanding the types and range of antibody responses and epitopes targeted on designed immunogens is a critical component of the feedback loop. Thus, next generation sequencing (NGS) of baseline repertoires and the antigen-specific repertoire post vaccination is a critical component of vaccine design in order to monitor germline usage and extent of somatic hypermutation.

In addition to sequence-level understanding of antibody responses, knowledge of which epitopes are targeted and the structural details of the interaction sites is critical. While traditional serology using scanning mutagenesis, viral neutralization and monoclonal antibody isolation continues to be valuable, new assays are emerging that further accelerate this process. One approach is deep mutational scanning. The combination of deep mutational scanning, viral growth in the presence of antibodies, and NGS of surviving viruses enables mapping of epitopes and functional mutations required for recognition by antibodies [50^•^]. Single particle electron microscopy polyclonal epitope mapping (EMPEM) is another exciting and recently developed technique that is able to visualize the antibody response directly from serum [51^•^]. These EM images provide immediate feedback regarding on- and off-target antibody responses in a time-dependent manner and reveal potential differences in the responses between individual animals, modes of immunogen delivery, effects of adjuvants and so on. These data can then be utilized for immediate immunogen redesign, circumventing the need to conduct time-consuming viral mutant neutralization assays and isolation of monoclonal antibodies.

## Conclusions

The field of structure-based vaccine design is rapidly maturing and can be applied to a wide variety of pathogens when antibodies with desirable properties are discovered and structures are then solved in complex with their antigens. These are the starting points for generating immunogens, or series of immunogens, that immunofocus antibody responses to sites of vulnerability. Excitingly, new antigenic targets are still being uncovered, providing new opportunities to apply this rational approach. For example, in influenza virus, very recent studies have ‘rediscovered’ neuraminidase (NA) as a promising target [52^••^,^53^,^54^]. Furthermore, a newly identified class of epitopes on HA, namely those at the interface between the HA1 head subunits, are have also been found to be a target for broad protective activity [55, 56, 57]. Both examples demonstrate that we should keep searching for new sites of vulnerability that can be exploited for immunogen design using the approaches described above.

While our review was limited to RSV, influenza, and HIV, many similar efforts are ongoing to create protein subunit vaccines to other pathogens such as Ebola, Lassa, Dengue, Coronaviruses, and others. Not surprisingly, much effort is currently being focused on producing a COVID-19 vaccine based on the prefusion structure of the spike (S) protein on the viral surface. Many of the lessons learned from other pathogens can be directly applied to SARS-CoV-2 and progress is proceeding at an unprecedented pace. Remarkably, the first structures of the COVID-19 S protein trimer took only a few weeks to produce after the sequence became available [58].

We are now on the verge of molecular control of immune responses using rationally designed immunogens, and we have the tools to probe such responses in humans, thereby circumventing the need for large empirical efficacy trials. Importantly, many of these concepts are currently being tested in humans as noted above and delineated in Table 1. As technology continues to improve, atomic-level knowledge of antibody-antigen recognition and modes of interaction remains the linchpin of immunogen design and should provide opportunities for novel vaccines beyond those for pathogens including cancer, autoimmunity, and neurodegeneration.

**Table 1:** Ongoing or recently completed human clinical trials testing neutralizing antibody potential and structure-based vaccine design concepts

Clinical trial #	Pathogen	Primary outcome
NCT03049488	RSV	Evaluate stabilized RSV pre-F vaccine for induction of neutralizing antibodies
NCT03300050	Influenza	Evaluate chimeric HA trimers for immuno-focusing on stalk epitope
NCT03814720	Influenza	Evaluate mini HA trimers for immuno-focusing on stalk epitope
NCT02825797	HIV	Evaluate the ability of a bnAb combination to suppress viremia in HIV positive individuals
NCT02716675	HIV	Evaluate the ability of a bnAb monotherapy to prevent HIV acquisition
NCT02568215	HIV	Evaluate the ability of a bnAb monotherapy to prevent HIV acquisition
NCT03699241	HIV	Evaluate stabilized HIV Env trimers for induction of neutralizing antibodies
NCT04177355	HIV	Evaluate stabilized HIV Env trimers for induction of neutralizing antibodies
NCT03783130	HIV	Evaluate stabilized HIV Env trimers for induction of neutralizing antibodies
NCT03547245	HIV	Evaluate germline targeting immunogen to prime and expand rare B cell bnAb precursors

## Conflict of interest statement

Nothing declared.

## References and recommended reading

Papers of particular interest, published within the period of review, have been highlighted as:

• of special interest
•• of outstanding interest
